# Supplementary material for: General decapping activators target different subsets of inefficiently translated mRNAs
Source: eLife. 2018 Dec 6;7:e34409. doi: 10.7554/eLife.34409 (PMC6300357; doi:10.7554/eLife.34409)
Supplement: Supplementary File 1. [file elife-34409-supp1.docx]

**Supplementary file 1. Yeast strains used in this study**

| **Name** | **Genotype** |
| --- | --- |
|  |  |
| HFY114(W303) | *MAT*a *ade2-1 his3-11,15 leu2-3,112 trp1-1 ura3-1 can1-100* |
| SYY2385 | *MATa ade2-1 his3-11,15 leu2-3,112 trp1-1 ura3-1 can1-100 dcp2-N245::KanMX6* |
| SYY2887 | *MATa ade2-1 his3-11,15 leu2-3,112 trp1-1 ura3-1 can1-100 dcp2-N245::KanMX6 xrn1::ADE2* |
| SYY2889 | *MATa ade2-1 his3-11,15 leu2-3,112 trp1-1 ura3-1 can1-100 dcp2-N245::KanMX6 ski2::URA3* |
| SYY2893 | *MATa ade2-1 his3-11,15 leu2-3,112 trp1-1 ura3-1 can1-100 dcp2-N245::KanMX6 ski7::URA3* |
| SYY2750 | *MATa ade2-1 his3-11,15 leu2-3,112 trp1-1 ura3-1 can1-100 dcp2-E153Q-N245::KanMX6* |
| SYY2897 | *MATa ade2-1 his3-11,15 leu2-3,112 trp1-1 ura3-1 can1-100 dcp2-E153Q-N245::KanMX6 xrn1::ADE2* |
| SYY2755 | *MATa ade2-1 his3-11,15 leu2-3,112 trp1-1 ura3-1 can1-100 dcp2-E198Q-N245::KanMX6* |
| SYY2901 | *MATa ade2-1 his3-11,15 leu2-3,112 trp1-1 ura3-1 can1-100 dcp2-E198Q-N245::KanMX6 xrn1::ADE2* |
| SYY2674 | *MATa ade2-1 his3-11,15 leu2-3,112 trp1-1 ura3-1 can1-100 pat1::KanMX6* |
| SYY2680 | *MATa ade2-1 his3-11,15 leu2-3,112 trp1-1 ura3-1 can1-100 lsm1::KanMX6* |
| SYY2686 | *MATa ade2-1 his3-11,15 leu2-3,112 trp1-1 ura3-1 can1-100 dhh1::KanMX6* |
| SYY2859 | *MATa ade2-1 his3-11,15 leu2-3,112 trp1-1 ura3-1 can1-100 pat1::KanMX6 ski2::URA3* |
| SYY2862 | *MATa ade2-1 his3-11,15 leu2-3,112 trp1-1 ura3-1 can1-100 lsm1::KanMX6 ski2::URA3* |
| SYY2865 | *MATa ade2-1 his3-11,15 leu2-3,112 trp1-1 ura3-1 can1-100 dhh1::KanMX6 ski2::URA3* |
| HFY871 | *MATa ade2-1 his3-11,15 leu2-3,112 trp1-1 ura3-1 can1-100 upf1::HIS3* |
| SYY2700 | *MATa ade2-1 his3-11,15 leu2-3,112 trp1-1 ura3-1 can1-100 upf1::HIS3 dhh1::ADE2* |
| HFY1067 | *MATa ade2-1 his3-11, 15 leu2-3, 112 trp1-1 ura3-1 can1-100 dcp1::URA3* |
| CFY1016 | *MATa ade2-1 his3-11,15 leu2-3,112 trp1-1 ura3-1 can1-100 dcp2::HIS3* |
| HFY1080 | *MATa ade2-1 his3-11, 15 leu2-3, 112 trp1-1 ura3-1 can1-100 xrn1::ADE2* |
| CFY25 | *MATa ade2-1 his3-11,15 leu2-3,112 trp1-1 ura3-1 can1-100 edc3::URA3* |
| SYY2352 | *MATa ade2-1 his3-11,15 leu2-3,112 trp1-1 ura3-1 can1-100 scd6::KanMX6* |
| HFY1170 | *MATa ade2-1 his3-11,15 leu2-3,112 trp1-1 ura3-1 can1-100 ski2::URA3* |
| SYY17 | *MATa ade2-1 his3-11,15 leu2-3,112 trp1-1 ura3-1 can1-100 ski7::URA3* |
| SYY21 | *MATa ade2-1 his3-11,15 leu2-3,112 trp1-1 ura3-1 can1-100 ski2::URA3* *ski7::ADE2* |
| SYY11 | *MATa ade2-1 his3-11, 15 leu2-3, 112 trp1-1 ura3-1 can1-100 rpb1-1* |
| SYY2959 | *MATa ade2-1 his3-11, 15 leu2-3, 112 trp1-1 ura3-1 can1-100 pat1::URA3 rpb1-1* |
| SYY2965 | *MATa ade2-1 his3-11, 15 leu2-3, 112 trp1-1 ura3-1 can1-100 lsm1::URA3 rpb1-1* |
| SYY2971 | *MATa ade2-1 his3-11, 15 leu2-3, 112 trp1-1 ura3-1 can1-100 dhh1::URA3 rpb1-1* |
| SYY150 (BY4741) | *MATa hisΔ1 leu2Δ0 met15Δ0 ura3Δ0* |
| SYY2930 | *MATa hisΔ1 leu2Δ0 met15Δ0 ura3Δ0 PAT1-TAP::KanMX6* |
| SYY2931 | *MATa hisΔ1 leu2Δ0 met15Δ0 ura3Δ0 LSM1-TAP::KanMX6* |
| SYY2932 | *MATa hisΔ1 leu2Δ0 met15Δ0 ura3Δ0 DHH1-TAP::KanMX6* |
| SYY2938 | *MATa hisΔ1 leu2Δ0 met15Δ0 ura3Δ0 PAT1-TAP::KanMX6 lsm1::URA3* |
| SYY2941 | *MATa hisΔ1 leu2Δ0 met15Δ0 ura3Δ0 PAT1-TAP::KanMX6 dhh1::URA3* |
| SYY2944 | *MATa hisΔ1 leu2Δ0 met15Δ0 ura3Δ0 LSM1-TAP::KanMX6 pat1::URA3* |
| SYY2947 | *MATa hisΔ1 leu2Δ0 met15Δ0 ura3Δ0 LSM1-TAP::KanMX6 dhh1::URA3* |
| SYY2950 | *MATa hisΔ1 leu2Δ0 met15Δ0 ura3Δ0 DHH1-TAP::KanMX6 pat1::URA3* |
| SYY2953 | *MATa hisΔ1 leu2Δ0 met15Δ0 ura3Δ0 DHH1-TAP::KanMX6 lsm1::URA3* |
