# Supplementary material for: General decapping activators target different subsets of inefficiently translated mRNAs
Source: eLife. 2018 Dec 6;7:e34409. doi: 10.7554/eLife.34409 (PMC6300357; doi:10.7554/eLife.34409)
Supplement: Supplementary File 2. [file elife-34409-supp2.docx]

**Supplementary file 2. Plasmids used in this study**

| **Name** | **Allele** | **Description** |
| --- | --- | --- |
| HFE2095 | Bs-Ks-*xrn1::ADE2* | Contains the *xrn1::ADE2* null allele as a *NotI-SalI*  fragment |
| HFE2289 | Bs-Ks-*ski2::URA3* | Contains the *ski2::URA3* null allele as a *NotI-SalI*  fragment |
| HFSE26 | Bs-Ks-*ski7::URA3* | Contains the *ski7::URA3* null allele as a *NotI-SalI* fragment |
| HFSE28 | Bs-Ks-*ski7::ADE2* | Contains the *ski7::ADE2* null allele as a *NotI-SalI* fragment |
| HFSE1387 | Bs-Ks-*dhh1::ADE2* | Contains the *dhh1::ADE2* null allele as a *NotI-SalI* fragment |
| HFSE1066 | Bs-Ks-*scd6:: KanMX6* | Contains the *scd6:: KanMX6* null allele as a *NotI-SalI* fragment |
| HFSE1364 | Bs-Ks-*pat1::URA3* | Contains the *pat1::URA3* null allele as a *NotI-SalI* fragment |
| HFSE1380 | Bs-Ks-*lsm1::URA3* | Contains the *lsm1::URA3*  null allele as a *NotI-SalI* fragment |
| HFSE1366 | Bs-Ks-*dhh1::URA3* | Contains the *dhh1::URA3* null allele as a *NotI-SalI* fragment |
| HFSE1147 | Bs-Ks-*dcp2-N245-KanMX6* | Described previously in He and Jacobson (2015) |
| HFSE1581 | Bs-Ks-*dcp2-E153Q-N245-KanMX6* | Contains the *dcp2-E153Q-N245* allele as a *NotI-XhoI* fragment, same as HFSE1147 but contains glutamic acid to glutamine change at codon position 153 |
| HFSE1583 | Bs-Ks-*dcp2-E198Q-N245-KanMX6* | Contains the *dcp2-E198Q-N245* allele as a *NotI-XhoI* fragment, same as HFSE1147 but contains glutamic acid to glutamine change at codon position 198 |
| HFSE1545 | pRS315-*HA*-*dcp2-N245* | Contains an N-terminal triple HA-tagged *dcp2-N245* allele as a 2.1kb *XbaI-SalI* fragment |
| HFSE1624 | pRS315-*HA*-*dcp2-E153Q-N245* | Contains an N-terminal triple HA-tagged *dcp2-E153Q-N245* allele as a 2.1kb *XbaI-SalI* fragment |
| HFSE1626 | pRS315-*HA*-*dcp2-E198Q-N245* | Contains an N-terminal triple HA-tagged *dcp2-E198Q-N245* allele as a 2.1kb *XbaI-SalI* fragment |
