# Supplementary material for: General decapping activators target different subsets of inefficiently translated mRNAs
Source: eLife. 2018 Dec 6;7:e34409. doi: 10.7554/eLife.34409 (PMC6300357; doi:10.7554/eLife.34409)
Supplement: Supplementary File 3. [file elife-34409-supp3.docx]

**Supplementary file 3. Oligonucleotides used in this study**

| **Name** | **Sequences** |
| --- | --- |
|  |  |
| CIT2-1-F | ATGACAGTTCCTTATCTAAATTCAAACAGA |
| CIT2-500-R | GCAGTTACAGCAATAGAGAATTGAGCCATT |
| SDS23-1-F | ATGCCTCAAAATACAAGACACACGTCCATC |
| SDS23-500-R | ATCTTGTCGTTGCTCACCTTGATCCTGTTT |
| HOS2-1-F | ATGTCTGGAACATTTAGTTATGATGTGAAA |
| HOS2-500-R | CCAGATGGACTATTCTTTTTTGCATGATGA |
| PYK2-1-F | ATGCCAGAGTCCAGATTGCAGAGACTAGCT |
| PYK2-500-R | ACCCTTAAATTAGATTCGTCAATGATTTGG |
| DIF1-1-F | ATGGACGCACAACTGGAATGGGCAAGCAGC |
| DIF1-400-R | AAAGTCTTCTCTTGGATCCATTAACCATTG |
| AGA1-1-F | ATGACATTATCTTTCGCTCATTTTACCTAC |
| AGA1-500-R | GAGATTATAGAGGCACTTGATGGTTCAATG |
| BUR6-1-F | ATGGCAGATCAAGTACCAGTTACAACACAA |
| BUR6-429-R | TCAGGCACTCTCTTCCTCCGGTTGTGTTTGG |
| LSM3-1-F | ATGGAGACACCTTTGGATTTATTGAAACTC |
| LSM3-270-R | TTATATCTCCACTGCGCCATCGTCATCTTC |
| HXT6-1-F | ATGTCACAAGACGCTGCTATTGCAGAGCAA |
| HXT6-470-R | ATACCGATGATGTAGATGACAACAACGACA |
| GPH1-1F | ATGCCGCCAGCTAGTACTAGTACTACCAAT |
| GPH1-500-R | TCATCCAAAGCCCCTTTAATCATTTCTCTT |
| HXK1-60-F | AAGGAATTGATGGATGAAATTCATCAGTTG |
| HXK1-480-R | TGGGTACGAGAAGGTGAAACCTAATGGTAA |
| CHA1-1-F | ATGTCGATAGTCTACAATAAAACACCATTA |
| CHA1-500-R | TGTTGCGATTTCAAATCTTGTACTATTTCA |
| RTC3-1-F | ATGTCTACTGTAACCAAATACTTTTACAAG |
| RTC3-336-R | TCAATTGTAGGCTTTGGTTCCGGCGTTACC |
| NQM1-481-F | AAGCATGGTATTCATTGTAATATGACATTA |
| NQM1-1002-R | TCACATTTTTTCTTCAACCAGTTTGTACAG |
| PGM2-1201-F | TTGAACATCTTGGCCATTTACAACAAGCAT |
| PGM2-1710-R | TTAAGTACGAACCGTTGGTTCTTCAGTTCC |
| TMA10-1-F | ATGACCAGAACTAGCAAATGGACAGTCCAC |
| TMA10-260-R | TAGATGTGGTATTGTTGCAAATCAGAAAGC |
| GAD1-1-F | ATGTTACACAGGCACGGTTCTAAGCAGAAG |
| GAD1-480-R | CAACATGATTGCCTCACTAGAACCTGTGGT |
| SPG4-1-F | ATGGGTAGTTTTTGGGACGCATTCGCAGTA |
| SPG4-340-R | TTACTTTATTGTCGGGTTCCCCCCTCCTCA |
| MUP3-1-F | ATGGAACCGCTGCTTTTTAATAGTGGGAAA |
| MUP3-500-R | ACGATAGATCCCGTCAATGCATAGCCAGTT |
| GTT2-1-F | ATGAATGGCAGAGGTTTCCTGATTTACAA |
| GTT2-500-R | TCAAAATAATGCATTCCATGTAGGGCTTTG |
| RPP1A-1-F | ATGTCTACTGAATCCGCTTTGTCTTACGCC |
| RPP1A-321-R | CTAATCAAATAAACCGAAACCCATGTCGTC |
| TMA19-1-F | ATGATTATTTACAAGGATATCTTCTCTAAC |
| TMA19-500-R | ATCTTTTCTTCCACAATACCGTGCTTCCAG |
| GPD2-1-F | ATGCTTGCTGTCAGAAGATTAACAAGATAC |
| GPD2-500-R | GCACCCTTGATGGAGTGTAAAAGATCAGGA |
| YIL164C-F | AAGGGAGGAGTATGCTAAGTATCT |
| YIL164C-R | CTAAATAGGCCTAGCATCCACCGT |
| THI22-F | GATTATGTGAGAGTTTGCTGCGTC |
| THI22-R | GCGGTCCAGAAATTAGTTTCTAAT |
| EST1-F | GAATGTGTTC TGCGAATTAGATCA |
| EST1-R | AGGAGTATCTGGCACTTGGACGGT |
| TRP1-1-F | ATGTCTGTTATTAATTTCACAGGTAGTTCT |
| TRP1-675-R | CTATTTCTTAGCATTTTTGACGAAATTTGC |
| ALR2-1-F | ATGTCGTCCTTATCCACTTCATTTGATTCA |
| ALR2-500-R | TTGCATCTGTTACTTGACGTACCGGCAGGT |
| GDH1-F | GAAACTGGTATCACCTCCGAACAAGTCGC |
| GDH1-R | TTAAAATACATCACCTTGGTCAAACATAGC |
| ARL1-F | ATGGGTAACATTTTTAGTTCAATGTTTGAC |
| ARL1-R | CTATAACTGTTCCTCTTTTATAACATCAAT |
| DAL3-F | ATGGTGACCGTGGTGGCGGAGACATTGACG |
| DAL3-R | TTAGATGATAATACAAACGTCGCCATCGCT |
| YGL117W-F | ATGCAGCCAATTTCAATAAAAGATGTGGAA |
| YGL117W-R | TCATATAACCCTTCTATGAGTTATTTTAAG |
| RPS9A-Exon-F | GAGCCCCAAGAACATATTCCAAGACTTACT |
| RPS9A-Exon-R | TTATTCTTCATCGGCCTCATCAGCTTCATC |
| SUC2-F | TGAACACTGAATATCAAGCTAATCCAGAGA |
| SUC2-R | CTATTTTACTTCCCTTACTTGGAACTTGTC |
| CPA1-F | TATGATTACAGAATTCAAGATGTTGCTTCT |
| CPA1-R | TTAGAACAACACTCTTTCCTTGGCCAACTT |
| SER3-F | CCACAATTTGCTGCTATGAAGGATGGCGCT |
| SER3-R | TTAATATAGCAATCTAATTGAGATCTTAGC |
| HACI-I-662-F | CCGTGATTACGATGACCAGGAAACTACAGT |
| HAC1-I-913-R | CGGACAGTACAAGCAAGCCGTCCATTTCTT |
| HSP82-F | ACTCAATTGAAGGAATTCGAAGGTAAAACT |
| HSP82-R | CTAATCTACCTCTTCCATTTCGGTGTCAGC |
| XRN1-DS5 | CGCCACCGCAGAGCAAGTAACAACAGAGAC |
| XRN1-DS6 | ACTGCCTCGAGTCTGACGATAGAAGACCCT |
| SKI2-DS1 | AATTCTAGAATTATCTTCAACGACTGAGAAGAA |
| SKI2-DS2 | AGAGGATCCATAAATTAGTATTAGTACAGTAAA |
| SKI2-DS3 | AATGGATCCATAATCGATAGAGCTCATTTATTCTCAATGTGA |
| SKI2-DS4 | TAAGTCGACAATACCATTTTCGCCTATCTTACC |
| Ski7-1(ATG-up-500) | AATTGCGGCCGCAACTGGATATTGTAGCGCCTAGCG |
| Ski7-2’ (ATG-up) | CGAGGAGGTGGTCTTCGAAACTTAGGATCCCGGATCGATAATT |
| Ski7-3 (TAA-down) | AATTATCGATCCTACAACTAAGAAATTATACTAGGCA |
| Ski7-4 (TAA-down-500) | TGTTTTACTTCGTCTTGTACAGT TTCTGTCGACAATT |
| PAT1-DS5 | AATTTATCGGCTAACAACTGTGACAGTTGC |
| PAT1-DS6 | TGTAACCCACCACACATGCCATGGTGATCG |
| LSM1-DS5 | AGACGAAGACGATGAGATCATTGATCCTAG |
| LSM1-DS6 | TGACATTTCTAAATCTGCGTAATCTTAGCT |
| DHH1-DS1 | GATCGCGGCCGCTTCGTAAGAAAAAGGACAACACAATCTTAG |
| DHH1-DS2 | GATCCCATGGAGATCTTACTACTATTTTCTTTCTTGTCGTATTTTA |
| DHH1-DS3 | GATCCCATGGGAATTCAGAATATCTAAGAAAAAATAACTACTGTGG |
| DHH1-DS4 | GATCGTCGACATGAAACTGGGCAAGTGCACTTGAGCTCTT |
| DHH1-DS7 | AGATGGGCCAATAATAGTATGATATCGCCGTTA |
| DHH1-DS8 | TCATCTTGTCAGTTGAAATGAATAGTTTATTGTGG |
| SCD6-DS1 | GATCGCGGCCGCCACATCTTCTTGCTCTTCTTATATTTACCA |
| SCD6-DS2 | GATCGAATTCATCAGATCTTGCCTTGCTGCTGTTTTTCGATGAATGCTT |
| SCD6-DS3 | GATCGAATTCAATGATGTTTCTATGTAAATTAAGTATATC |
| SCD6-DS4 | GATCGTCGACTAACCAATTGGCCATCAAACTTTACGAAAA |
| DCP2-NheI-F | TTACGCTAGCATGTCACTGCCGCTACGACACGCATTGGAG |
| DCP2-N245-E1-R | GATCGAATTCATCTTCATTTTTTATTTGCCTCTGATGCCT |
